# The Association between Body Mass Index and Glycohemoglobin (HbA1c) in the US Population’s Diabetes Status

**DOI:** 10.3390/ijerph21050517

**Published:** 2024-04-23

**Authors:** Wenxue Lin

**Affiliations:** Department of Epidemiology and Biostatistics, College of Public Health, Temple University, Philadelphia, PA 19122, USA; wenxue.lin@temple.edu

**Keywords:** body mass index, glycated hemoglobin (HbA1c), diabetes, NHANES, prediabetic

## Abstract

Obesity, indicated by Body Mass Index (BMI), is a risk factor for type 2 diabetes. However, its association with glycated hemoglobin (HbA1c), a crucial indicator of blood-sugar control, may vary across different populations and disease statuses. Data from the National Health and Nutrition Examination Survey (NHANES) 2017–2018 were analyzed. Participants aged 18–79 years with complete information on BMI, diabetes status, and HbA1c were included (n = 4003). Linear regression models were used to assess the association between BMI and HbA1c, adjusting for demographic confounders, smoking status, alcohol consumption, and healthcare access. Among participants without diabetes, BMI was positively associated with HbA1c levels (coefficient: 0.015, 95% CI: 0.01, 0.02; *p*-value < 0.05), after adjusting for potential confounders. However, this association was not significant among those with diabetes (coefficient: −0.005, 95% CI: −0.05, 0.04; *p*-value > 0.1). Our findings suggest a differential relationship between BMI and HbA1c in individuals with and without diabetes. While BMI remains a significant predictor of HbA1c in non-diabetic individuals, its significance diminishes in those with diabetes.

## 1. Introduction

Diabetes is one of the chronic diseases that is responsible for severe complications such as kidney failure, stroke, retinopathy, neuropathy, blindness, and lower-limb amputation [1,2]. In 2014, there were more than 400 million people living with diabetes, and an estimated two million deaths as a result of diabetes and its complications [1]. In the United States alone, more than 30 million people are suffering from diabetes. Alarmingly, 20% of them do not even realize they have the disease [3]. Further, almost ten million people in the United States are in the stage of prediabetes (a serious potential health problem with blood-sugar levels higher than the normal range but not reaching the confirmative type 2 diabetes level). Meanwhile, 80% of them lacked awareness regarding the risk of being prediabetic or of not monitoring their blood-sugar fluctuations [3].

As the eighth leading cause of mortality in the United States, there are two types of diabetes: type 1, constituting approximately 5–10% of diabetes cases, and type 2, which accounts for most of the diabetes cases (90–95%) [3]. Type 1 diabetes stems from insulin deficiency, either due to the complete absence or scant production by the pancreas [1,3]. The lack of insulin leads to the accumulation of blood sugar in the bloodstream, rendering organs and cells unable to metabolize blood sugar without the help of insulin [3]. In type 2 diabetes, on the other hand, patients retain the capacity to secrete insulin; however, insulin resistance occurs where cells do not respond to the insulin signals, fostering blood-sugar accumulation, which further contributes to complications of diabetes, including damage to nerves, vision loss, and kidney failure [3]. The economic burden of diabetes is significant, with the total cost of diabetes in the United States surpassing 410 billion in 2022. People with diabetes face nearly triple the medical expenditures than people without diabetes [4].

Obesity, age (45 years or older), family history (having a parent, sister, or brother with type 2 diabetes), race/ethnicity (being African American, Hispanic or Latino, American Indian, or Alaska Native), and physical inactivity are risk factors for diabetes [2,5,6,7,8]. Body Mass Index (BMI kg/m^2^) has been widely used to indicate general adiposity, categorizing individuals as underweight (below 18.5), normal (18.5–24.9), overweight (25.0–29.9), and obese (30.0 and above) [2,9]. Further, several studies have indicated a strong association between BMI and the risk of diabetes, making BMI one of the reliable predictors of type 2 diabetes [10]. In addition to obesity and BMI, smoking behaviors also contribute to the increased risk of diabetes. Nicotine alters cells, and toxicants in cigarettes jeopardize cell function, leading to inflammation that impairs insulin response and elevates blood-sugar levels [11,12].

Glycated hemoglobin (HbA1c) is the most accurate and reliable (often considered gold standard) criterion for diagnosing diabetes mellitus (DM) [13]. Specifically, DM is diagnosed in patients with HbA1c levels greater than or equal to 6.5%, while HbA1c ranges from 5.7% to 6.4% indicate prediabetes [14]. Despite obesity being a well-known risk factor that contributes to diabetes and abnormal glucose control, limited studies have investigated the effect of changes in BMI on glycated hemoglobin. Therefore, the current study aims to assess the relationship between a single unit change in BMI and its impact on HbA1c. Further, our study seeks to investigate the differential impact of a one-unit change in BMI on HbA1c based on diabetes status (people with diabetes vs. people without diabetes). By addressing this gap, our study not only enhances understanding regarding the impact of a single-unit change in BMI on HbA1c levels, but also underscores the crucial roles played by BMI in maintaining a healthy weight and glycated hemoglobin levels.

## 2. Materials and Methods

### 2.1. NHANES Data

This study used data from the National Health and Nutrition Examination Survey (NHANES) 2017–2018. The NHANES program, initiated in the 1960s, has been implemented for a few surveys targeting on a wide range of health topics, including smoking, sexual practices, drug use, dietary intake, physical fitness, obesity, diabetes, cardiovascular disease, chronic disease, and sexually transmitted disease, to name a few [15]. The NHANES is administered by the National Center for Health Statistics (NCHS) and the Centers for Disease control and Prevention (CDC), providing critical, valid, and reliable key information for the health of the United States’ (U.S.’s) adults [15]. It is important to note that the NHANES sampled civilians of the noninstitutionalized U.S. population, excluding individuals in custody or institutional settings from the study [15]. Health interviews and measurements were conducted in respondents’ homes and mobile examination centers, respectively [15]. The NHANES employed a complex, four-stage sampling procedure, involving the selection of primary sampling units (PSUs) (Step 1), choosing segments within the counties (Step 2), selecting dwelling units (DUs) (Step 3), and selecting individuals within households (Step 4) [16]. More details about the NHANES such as the study design, sampling weights, survey methods, analytic guidelines, biospecimen program, questionnaires, datasets, related documentation, and estimation and weighting can be found on the Centers for Disease control and Prevention website: https://www.cdc.gov/nchs/nhanes/about_nhanes.htm (accessed on 15 March 2024).

### 2.2. Study Population

Out of the 9254 participants who completed the NHANES 2017–2018 study, we excluded participants younger than 17 years or older than 80 years (n = 3825). In addition, participants with missing demographics information (gender, race/ethnicity, education, or annual household income), smoking status, alcohol consumption, and place to go for healthcare were excluded. Those with incomplete data on BMI, diabetes status, and glycohemoglobin (HbA1c; %) were also excluded from the study (n = 1426). A total of 4003 participants were eligible for the study analysis of diabetes vs. non-diabetes. Among the eligible participants, 11.3% (weighted proportion; unweighted n = 625) were participants with diabetes, and 88.7% (weighted proportion; unweighted n = 3378) were participants without diabetes.

### 2.3. Measures

The current study included a comprehensive set of variables, including demographic information (gender, race/ethnicity, education, and annual household income), smoking status, alcohol consumption, and healthcare accessibility. Gender (male vs. female), race/ethnicity (Hispanic, Non-Hispanic White, Non-Hispanic Black, and Others), education attainment (less than a high school diploma vs. more than a high school diploma, e.g., some college graduates or above), and annual household income (USD 0 to USD 54,999 vs. more than or equal to USD 50,000) were treated as categorical variables. Healthcare-accessibility information was obtained from the Hospital Utilization and Access to Care (HUQ_J) questionnaire: “Is there a place that you usually go when you are sick, or you need advice about your health?”. The responses were categorized as “Yes, at least one place” or “No place”, with combined original responses of “Yes” and “There is more than one place” grouped under “Yes, at least one place”.

We used “Smoking-Cigarette Use”-questionnaire data to assess participants’ smoking statuses. Participants were categorized into three different groups based on their smoking status: 1. Non-smokers; defined as people who answered “No” to the question “Have you smoked at least 100 cigarettes in your entire life?”; 2. Former smokers, defined as people who answered “Yes” to the question “Have you smoked at least 100 cigarettes in your entire life?” but responded “No” to the second question “Do you now smoke a cigarette?”; and 3. Current cigarette smokers, comprising those who answered “Yes” to both questions (smokers who had smoked at least 100 cigarettes in their life and currently smoke cigarettes) [17,18]. Similarly, alcohol-drinking status was determined by the “Alcohol use” questionnaire, where participants were grouped as alcohol drinkers if they reported “Yes” to the question “The next questions are about drinking alcoholic beverages. Included are liquor (such as whiskey or gin), beer, wine, wine coolers, and any other type of alcoholic beverage. In {your} entire life, {have you/has he/has she} had at least 1 drink of any kind of alcohol, not counting small tastes or sips? By a drink, I mean a 12 oz. beer, a 5 oz. glass of wine, or one and a half ounces of liquor”. Those who answered “No” were marked as non-drinkers.

Body Mass Index (BMI, kg/m^2^) information was obtained from Body-measurement data. BMI was used as both categorical and continuous variables in our study to investigate the association between a single-unit change in BMI and its effect on HbA1c. For categorical BMI classification, participants were categorized as underweight (BMI less than 18.5), normal weight (BMI between 18.5 and 24.9), and overweight (BMI ≥ 25.0 but less than 29.9), or obese (BMI ≥ 30.0) [19,20]. Diabetes status was determined using the “Diabetes” questionnaire, specifically, “The next questions are about specific medical conditions. {Other than during pregnancy, {have you}/{Have you}} ever been told by a doctor or health professional that {you have/{he/she} has} diabetes or sugar diabetes?”. Participants were classified as having diabetes if they answered “Yes”. Those who answered “No” were classified as No diabetes. Data on glycohemoglobin (%; HbA1c) were extracted from “Glycohemoglobin” laboratory data. Detailed descriptions of the laboratory methods used in glycohemoglobin assessments can be accessed from the following: https://wwwn.cdc.gov/nchs/nhanes/search/datapage.aspx?Component=Laboratory&CycleBeginYear=2017 (16 March 2024).

### 2.4. Statistical Analysis

The characteristics of participants were compared by diabetes status using Rao-Scott Chi-square tests for categorical variables and *t*-tests for continuous variables [17,21,22,23]. Simple and multivariable linear-regression models were used to assess the unadjusted and covariate-adjusted relationship between BMI and glycohemoglobin (HbA1c). Model 1 (weighted simple linear regression) examined the relationship between BMI and HbA1c without adjusting for any covariates, presenting coefficients, *p*-values, and a 95% Confidence Interval (CI). Model 2 (weighted multiple linear regression) assessed the association between BMI and HbA1c, adjusting for demographic covariates (gender, race/ethnicity, education, annual household income), with coefficients, *p*-values, and a 95% Confidence Interval provided. Model 3 (weighted multiple linear regression) analyzed the association between BMI and HbA1c after further adjustment for smoking status, alcohol consumption, and healthcare access, in addition to demographic covariates (gender, race/ethnicity, education, annual household income), with coefficients, *p*-values, and a 95% Confidence Interval reported.

All three models (Model 1, 2, and 3) were evaluated across the entire study population (participants with and without diabetes), as well as within subgroups of individuals without diabetes and those with diabetes. Our analysis approach allowed for a comprehensive assessment of the impact of a single-unit change in BMI on HbA1c across different populations based on diabetes status.

All statistical analyses and visualizations were performed using R statistical software (version 4.3.3). Appropriate R packages (e.g., survey packages and survey design) were loaded, used, and created for statistical analysis, incorporating appropriate weights, strata, and clustering variables to account for the complex sampling design of NHANES [24,25,26]. All tests were two-sided, with a significance level set at 0.05.

## 3. Results

Table 1 presents the characteristics of the study participants, stratified by diabetes status. Of all eligible participants, 11.3% (weighted) had diabetes, while 88.7% (weighted) had no diabetes. Participants with diabetes tended to be older (55.9 vs. 45.3), male (56.8% vs. 47.8%), had at least one routine healthcare access (91.8% vs. 79.1%), had obesity (65.9% vs. 40.3%), were former smokers (38.2% vs. 23.1%), had a higher BMI (33.9 vs. 29.3), and had a higher amount of glycohemoglobin (7.3 vs. 5.5) compared to participants without diabetes (*p* < 0.05). There were no significant differences in race/ethnicity, education attainment, annual household income, and alcohol-drinker status between participants with diabetes and participants without diabetes (*p* > 0.05).

Table 2 presented the linear-regression analysis examining the relationship between BMI and glycohemoglobin (HbA1c) within the total study population. In Model 1, for each 1 kg/m^2^ increase in BMI, there was a corresponding increase in HbA1c of 0.028% (95% CI: 0.023, 0.034; *p*-value < 0.001). In model 2, after adjusting for age, gender, race/ethnicity, education, and annual household income, each additional increase in BMI was associated with a 0.025% increase in HbA1c (95% CI: 0.019, 0.031; *p*-value < 0.001). Further, in model 2, we observed that being NH-White was associated with a lower HbA1c level compared to being Hispanic (coefficient: −0.17, 95% CI: −0.31, −0.01; *p* = 0.04). In Model 3, where we further adjusted for smoking status, alcohol-drinking status, and healthcare access, BMI remained a significant indicator for HbA1c (coefficient: 0.025, 95% CI: 0.017, 0.034; *p* = 0.002).

**Figure 1:**
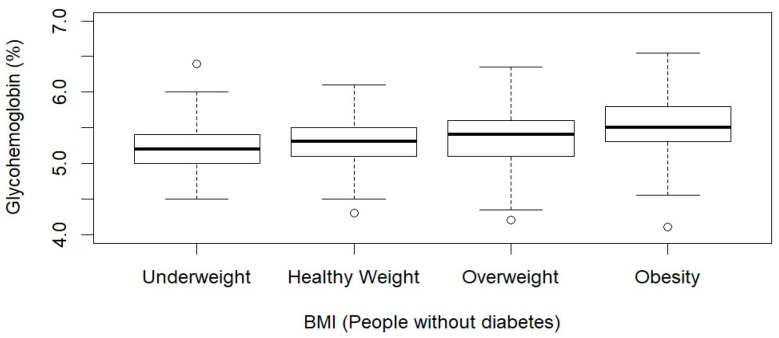
Boxplot of BMI and HbA1c among participants without diabetes.

Table 3 presented the findings of the linear-regression analysis conducted among the subpopulation without diabetes. In Model 1, each 1 kg/m^2^ increase in BMI was associated with a 0.016% (95% CI: 0.012, 0.019; *p*-value < 0.001) increase in HbA1c. In Model 2, after adjusting for age, gender, race/ethnicity, education, and annual household income, each additional increase in BMI corresponded to a 0.015% (95% CI: 0.011, 0.018; *p*-value < 0.001) increase in HbA1c levels. Further, in Model 2, we observed that NH-White individuals had lower HbA1c levels (coefficient: −0.11, 95% CI: −0.18, −0.04; *p* = 0.006), while NH-Black individuals had higher HbA1c levels (coefficient: 0.08, 95% CI: 0.01, 0.14; *p* = 0.017) compared to Hispanic. In Model 3, after further adjustment for smoking status, alcohol consumption, and healthcare access, NH-White participants maintained lower HbA1c levels (coefficient: −0.13, 95% CI: −0.22, −0.03; *p* = 0.02) compared to Hispanic individuals. Similarly, BMI remained a significant predictor of HbA1c (coefficient: 0.015, 95% CI: 0.01, 0.02; *p* = 0.002). Figure 1 demonstrates the distribution of HbA1c levels by BMI status. The median for HbA1c showed an increasing trend as BMI status progressed from healthy weight to obesity.

Additionally, Table 4 reports the findings of a linear-regression analysis conducted among the subpopulation with diabetes. In Model 1, each 1 kg/m^2^ increase in BMI was associated with a 0.01% decrease in HbA1c, but it was not statistically significant (95% CI: −0.034, 0.024; *p*-value = 0.71). In Model 2, after adjusting for age, gender, race/ethnicity, education, and annual household income, each additional increase in BMI was still associated with a 0.01% decrease in HbA1c, and again, this was not statistically significant (95% CI: −0.04, 0.03; *p*-value = 0.68). In Model 3, further adjustment for smoking status, alcohol consumption, and healthcare access did not alter the non-significant association between BMI and HbA1c. BMI remained a non-significant indicator for HbA1c in this model (coefficient: −0.005, 95% CI: −0.05, 0.04; *p* = 0.74).

## 4. Discussion

BMI is a significant indicator for glycohemoglobin (HbA1c) across both the total population and the subpopulation without diabetes in all three models. Our study findings align with prior research, such as that of Nyamdorj et al., who underscored the strong relationship between BMI and diabetes development, emphasizing the critical roles played by BMI as an indicator for chronic metabolic disease [10]. Obesity and weight gain are risk factors for type 2 diabetes [27,28,29], while physical activity and weight management serve as effective preventive measures for diabetes in people with normal and impaired blood-sugar regulation [30]. However, the Centers for Disease Control and Prevention (CDC) data paint a concerning picture: as of 2017–2018, approximately 31% of US adults were overweight, and 42.4% were obese (including 9.2% of severe obesity, defined as a BMI of 40 or greater) [31]. Even more concerning is the upward trend in obesity and severe obesity prevalence from 1999 to 2000 to 2017 to 2018, with rates climbing from 30.5% to 42.4% for obesity and from 4.7% to 9.2% for severe obesity [31]. Conversely, less than 25% of US adults aged 18 or older met the recommended physical-activity guidelines, with more than 45% failing to meet any type of physical-activity guidelines [32]. Regarding gender-specific trends, 28.3% of men engaged in both aerobic and muscle-strengthening activities for at least 150 min per week, compared to 20.4% of women. However, the frequency of physical activity declines with age in both men and women [32]. Meta-analyses have indicated nearly a tripled risk of type 2 diabetes in overweight people and a sevenfold increase in obesity compared to those with a normal weight [33], emphasizing the important and close relationship between weight gain and type 2 diabetes risk [34,35]. Physical activity and weight control not only serve as crucial protection factors but also constitute one of the United States Healthy People 2030 Leading Health Indicators due to their potential to enhance health and prevent adverse health outcomes, including type 2 diabetes and cardiovascular disease [36,37]. Nonetheless, given the high prevalence of overweight, obesity, and inadequate physical activity, the continued lapses in weight control remain a pressing concern, exacerbating the severe public health issues associated with diabetes and imposing a heavy economic burden on medical expenditures for patients and the healthcare system.

In our study, we observed that compared to Hispanic participants, NH-White participants faced a lower risk of elevated HbA1c levels. We found that race/ethnicity had a stronger effect, especially within the subpopulation without diabetes, as NH-White participants had significantly lower coefficients, while NH-Black participants showed significantly higher increases in glycohemoglobin compared to Hispanic participants in Model 2 (Table 3). Even after adjusting for smoking status and alcohol-drinking status (Model 3, Table 3), the impact of having a NH-White race/ethnicity remained significant at *p* = 0.02. Certain racial and ethnic minorities are disproportionately affected by type 2 diabetes and prediabetes [38]. Hispanic or Latino people and African American people experienced significantly higher rates of diabetes [38].

However, we did not observe similar trends in BMI risk factors or race/ethnicity disparities among the subpopulation with diabetes. BMI was not a significant predictor for HbA1c in people with diabetes across all three models (Table 4). Further, there was no difference in HbA1c levels by race/ethnicity; although the coefficient for NH-White was −0.32, it was no longer statistically significant (*p* = 0.34). Among participants with diabetes, nearly 92% had access to healthcare facilities, suggesting that the majority may have received treatment, such as insulin therapy. This may partially explain the diminished effect of BMI on HbA1c levels observed in the subpopulation with disease. Overall, the relationship between BMI and HbA1c may be complex and multifaceted, particularly in the context of diabetes, where various physiological and lifestyle factors interact to influence blood-sugar control.

In addition to BMI, waist circumference or waist–height ratio (WHtR) have been widely used as another indicator of type 2 diabetes, since BMI may not accurately reflect fat distribution in the abdomen [2,9,39]. Therefore, waist circumference or waist–height ratio (WHtR) might better represent abdominal visceral adiposity. Further, Fan et al. found that abdominal adiposity indicators, such as waist circumference and its changes, showed a stronger association with type 2 diabetes than BMI alone [2]. Cigarette smokers with diabetes pose a significant public health concern since they face more challenges in insulin dosing and glucose control [14]. The nicotine and chemical toxicants keep damaging cell function, ultimately leading to insulin resistance [12], which further exacerbates diabetes and contributes to more negative health problems, for example, kidney disease, retinopathy, and peripheral neuropathy [40]. Improving and increasing awareness of the risks associated with nicotine products, including cigarettes, low-nicotine-content cigarettes, and addiction, may be urgent for the public, especially for smokers with diabetes [41,42,43,44,45,46]. Carbohydrates play a pivotal role in blood-sugar regulation and the development of type 2 diabetes. Typically, when individuals consume carbohydrate-rich foods, the digestive system breaks them down into sugar, which enters the bloodstream [47]. The pancreas then secretes insulin, allowing cells to absorb the blood sugar for energy or storage. However, in individuals with diabetes, muscle and other cells become resistant to insulin, resulting in persistently high blood-sugar levels. This condition, known as insulin resistance, progresses gradually over several years. The continuous strain on insulin-producing cells eventually leads to diminished insulin production, indicating the transition from prediabetes to type 2 diabetes [47,48]. Healthcare providers should continue to monitor BMI in all individuals, since it remains a significant predictor of HbA1c levels, particularly in those without diabetes. Healthcare professionals should adopt a more individualized approach to blood-sugar control, considering factors in addition to BMI, such as insulin resistance, dietary habits, and physical-activity levels. Given the complexity of blood-sugar control in individuals with diabetes, comprehensive care should involve different interventions, including medication management, lifestyle modifications, and the regular monitoring of HbA1c levels.

Our study has several limitations. BMI is an indicator for general adiposity; however, we did not account for waist circumference, waist–height ratio or changes in these abdominal-adiposity indices. Future studies may need to consider or incorporate both general- and abdominal-adiposity indices for the assessment of diabetes and HbA1c. We used data from the NHANES, and, therefore, the study results may not be generalizable to populations residing outside of the United States, since the NHANES only collects data within the US. Nevertheless, our study aligns with previous research conducted in China, where a similar close association was observed between obesity and a higher risk of diabetes [49]. Improved nutrition and physical activity are essential components of a healthy lifestyle and are critical in the prevention and management of type 2 diabetes [50,51]. Our study did not assess the impact of nutrition or physical activity, and future studies may need to further explore the association between dietary intake, BMI, and glycated hemoglobin. Many participants with diabetes had access to healthcare, and some of them might be using insulin, which could weaken the effect of BMI. However, we did not adjust for these potential factors (medication usage, insulin resistance, and disease duration) in our study. Further, we did not differentiate between type 1 and type 2 diabetes in our study, and the relationship between BMI and different types of diabetes might be stratified for comparison in future studies. With 11.6% of the US population suffering from diabetes, more than one-third (38%, approximately 98 million) of US adults have prediabetes (defined as HbA1c between 5.7 and 6.4% or fasting plasma-glucose values of 100 to 125 mg/dL) [48,52]. The lack of awareness of prediabetes exacerbates their health, as more than 80% of them are not even aware of being prediabetic [48]. Therefore, another potential future direction could be conducting a longitudinal study to assess the long-term effect of BMI, waist–height ratio, and changes in these general- and abdominal-adiposity indices on HbA1c among prediabetes, given the increasing prevalence and heavy burden of prediabetes.

## 5. Conclusions

Our findings suggest a differential relationship between BMI and HbA1c in individuals with and without diabetes. While BMI remains a significant predictor of HbA1c in non-diabetic individuals, its significance diminishes in those with diabetes. These results underscore the complexity of blood-sugar control by diabetes status.

## Figures and Tables

**Table 1:** Characteristics of participants by diabetes status, 2017–2018 NHANES sample.

	Diabetes N = 625 (11.3%)	Non-Diabetes N = 3378 (88.7%)	*p*-Value
Gender			**0.038**
Male	353 (56.8)	1597 (47.8)	
Female	272 (43.2)	1781 (52.2)	
Race/ethnicity *			0.85
Hispanic	96 (8.3)	479 (9.0)	
NH-White	209 (63.8)	1180 (63.6)	
NH-Black	146 (10.8)	758 (10.3)	
Others	174 (17.0)	961 (17.0)	
Education			0.093
<High school diploma	311 (44.5)	1405 (37.4)	
≥High school diploma	314 (55.5)	1973 (62.6)	
Place to go for healthcare			**<0.001**
At least one place	577 (91.8)	2622 (79.1)	
No place	48 (8.2)	756 (20.9)	
Annual household income			0.62
USD 0 to USD 54.999	391 (48.7)	1943 (46.6)	
≥USD 55,000	234 (51.3)	1435 (53.4)	
BMI (kg/m^2^)			**<0.001**
Underweight (below 18.5)	1 (0.1)	54 (1.50)	
Healthy Weight (18.5–24.9)	73 (8.8)	870 (26.3)	
Overweight (25.0–29.9)	181 (25.1)	1089 (32.0)	
Obesity (30.0 and above)	370 (65.9)	1365 (40.3)	
Cigarette-smoking status			**<0.001**
Non-smoker	302 (48.9)	1996 (58.2)	
Former smoker	229 (38.2)	713 (23.1)	
Current smoker	94 (12.9)	669 (18.6)	
Alcohol-drinker status			0.66
Non-drinker	55 (7.7)	324 (6.8)	
Alcohol drinker	570 (92.3)	3054 (93.2)	
Age, year	59.9 (0.9)	45.3 (0.7)	**<0.001**
BMI (kg/m^2^)	33.9 (0.7)	29.3 (0.2)	**<0.001**
Glycohemoglobin	7.3 (0.09)	5.5 (0.01)	**<0.001**

Data source: NHANES 2017–2018. Categorical variables: unweighted N (weighted %); continuous variables: N (weighted %). *p* value was calculated by the Rao–Scott x2 test and *t*-test for categorical variables and continuous variables, respectively. Bolded *p*-values indicated significance. * NH: Non-Hispanic.

**Table 2:** Regression parameters from weighted linear-regression models (total population).

	Coefficient (95% CI)	*p*-Value
Model 1 (Crude) *		
BMI	0.028 (0.023, 0.034)	<0.001
Model 2 (Adjusted)		
Gender		0.03
Male	[Reference]	
Female	−0.09 (−0.16, −0.01)	
Race/ethnicity		
Hispanic	[Reference]	
NH-White	−0.17 (−0.32, −0.01)	0.04
NH-Black	0.09 (−0.05, 0.23)	0.16
Others	0.02 (−0.13, 0.16)	0.76
BMI	0.025 (0.019, 0.031)	<0.001
Age	0.018(0.016, 0.021)	<0.001
Model 3 (Adjusted)		
Gender		0.14
Male	[Reference]	
Female	−0.08 (−0.22, 0.05)	
Race/ethnicity		
Hispanic	[Reference]	
NH-White	−0.18 (−0.39, 0.03)	0.07
NH-Black	0.09 (−0.09, 0.27)	0.23
Others	0.005 (−0.18, 0.19)	0.93
BMI	0.025 (0.017, 0.034)	0.002
Age	0.018 (0.016, 0.021)	<0.001

* Model 1: crude model without any confounders adjusted. Model 2: adjusted for age, gender, race/ethnicity, education, and annual household income. Model 3: adjusted for Model 2 confounders + smoking status, drinking status, and healthcare access.

**Table 3:** Regression parameters from weighted linear-regression models (people without diabetes).

	Coefficient (95% CI)	*p*-Value
Model 1 (Crude) *		
BMI	0.016 (0.012, 0.019)	<0.001
Model 2 (Adjusted)		
Gender		0.59
Male	[Reference]	
Female	0.01 (−0.04, 0.06)	
Race/ethnicity		
Hispanic	[Reference]	
NH-White	−0.11 (−0.18, −0.04)	0.006
NH-Black	0.08 (0.02, 0.14)	0.017
Others	0.02 (−0.06, 0.1)	0.56
BMI	0.015 (0.011, 0.018)	<0.001
Age	0.011 (0.010, 0.012)	<0.001
Model 3 (Adjusted)		
Gender		0.49
Male	[Reference]	
Female	0.03 (−0.08, 0.13)	
Race/ethnicity		
Hispanic	[Reference]	
NH-White	−0.13 (−0.22, −0.03)	0.02
NH-Black	0.07 (−0.01, 0.16)	0.08
Others	0.01 (−0.09, 0.11)	0.80
BMI	0.015 (0.01, 0.02)	0.002
Age	0.011 (0.01, 0.014)	<0.001

* Model 1: crude model without any confounders adjusted. Model 2: adjusted for age, gender, race/ethnicity, education, and annual household income. Model 3: adjusted for Model 2 confounders + smoking status, drinking status, and healthcare access.

**Table 4:** Regression parameters from weighted linear regression models (people with diabetes).

	Coefficient (95% CI)	*p*-Value
Model 1 (Crude) *		
BMI	−0.01 (−0.034, 0.024)	0.71
Model 2 (Adjusted)		
Gender		0.01
Male	[Reference]	
Female	−0.32 (−0.54, −0.1)	
Race/ethnicity		
Hispanic	[Reference]	
NH-White	−0.32 (−1.1, 0.4)	0.34
NH-Black	0.25 (−0.56, 1.05)	0.49
Others	−0.24 (−0.97, 0.49)	0.45
BMI	−0.01 (−0.04, 0.03)	0.68
Age	−0.013 (−0.03, 0.002)	0.08
Model 3 (Adjusted)		
Gender		0.06
Male	[Reference]	
Female	−0.36 (−0.73, 0.02)	
Race/ethnicity		
Hispanic	[Reference]	
NH-White	−0.31 (−1.3, 0.69)	0.40
NH-Black	0.27 (−0.80, 1.34)	0.48
Others	−0.24 (−1.2, 0.7)	0.48
BMI	−0.005 (−0.05, 0.04)	0.74
Age	−0.01 (−0.03, 0.01)	0.15

* Model 1: crude model without any confounders adjusted. Model 2: adjusted for age, gender, race/ethnicity, education, and annual household income. Model 3: adjusted for Model 2 confounders + smoking status and drinking status and healthcare access.

## Data Availability

The NHANES data is publicly available on the CDC website: https://www.cdc.gov/nchs/nhanes/index.htm (accessed on 15 March 2024).
